# Global uncorrected refractive error and presbyopia: the size of the problem

**Published:** 2024-05-15

**Authors:** Andrew Bastawrous, Jordan Kassalow, Elanor Watts

**Affiliations:** 1 Professor Global Eye Health: International Centre for Eye Health, London School of Hygiene and Tropical Medicine, Co-Founder & CEO: Peek Vision, London, UK, and Co-Founder: Vision Catalyst Fund.; 2 Founder & Vice Chairman: VisionSpring, New York, USA and Co-founder & Chairman, EYElliance.; 3 Research Consultant: Peek Vision, UK and Ophthalmology Registrar: Tennent Institute of Ophthalmology, Glasgow, UK.

**Millions are struggling to learn, be healthy, and earn a living because they don't have spectacles.**

**Figure:**
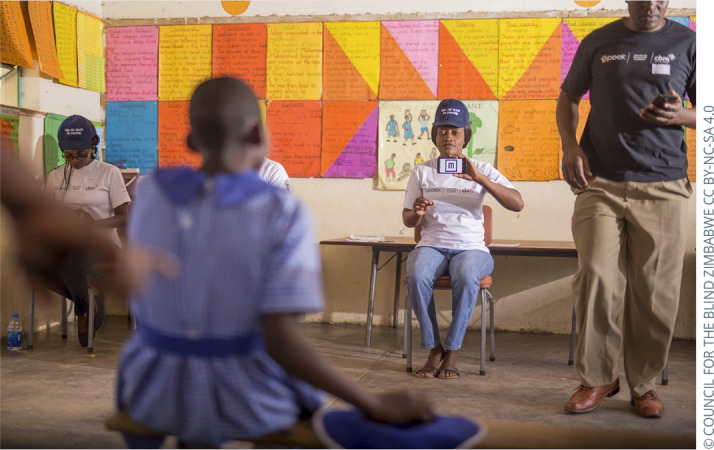
A child is screened for refractive error in her school. zimbabwe

Uncorrected refractive error is often ignored in the realm of global health priorities, yet its substantial impact on the economic and personal wellbeing of individuals and societies worldwide is undeniable. Myopia (shortsightedness), hyperopia (farsightedness), and astigmatism, grouped together under the term ‘refractive error’, cause blurred distance and/or near vision, and presbyopia (age-related loss of accommodation) causes blurred near vision. Although presbyopia has a different mechanism to the other refractive errors, all these conditions can be corrected using spectacles or contact lenses. For the purposes of this article, therefore, we will use the term refractive error to refer to refractive error and presbyopia.

Unless refractive errors are corrected (using spectacles, contact lenses, or otherwise), the children and adults affected will experience difficulties in tasks that are crucial for daily living, education, and employment.

In this article, we summarise the estimated prevalence, the populations affected, and the implications if refractive error and optical services are not extended to everyone who needs them. We aim to equip policy makers and refractive error care providers alike to advocate for the resources required to tackle this global issue.

## How big is the problem?

The estimates of global magnitude vary widely, due to relatively limited primary data and the different modelling assumptions made (see panel).

- **Blindness due to uncorrected refractive error** (defined as distance visual acuity worse than 3/60): the current estimate is 3.7 million^1^,^2^
- **Moderate or severe distance vision impairment due to refractive error** (defined as distance visual acuity worse than 6/18 but equal to or better than 3/60): estimates range from 123.7 million^3^ to 157 million^1^,^2^
- **Near vision impairment due to presbyopia** (defined as near visual acuity worse than N6 at 40 cm): estimates range from 510 million^1^,^2^ to 826 million.^3^,^4^

In total, up to 1 billion people worldwide, predominantly in Africa and Asia, are blind or have vision impairment because they do not have the spectacles they need.^1^,^2^,^3^,^4^

The prevalence and distribution of near and distance vision impairment due to uncorrected refractive error is expected to change significantly in coming decades, due in part to the rise of myopia, most rapidly in East Asia, and to a rise in presbyopia, due to population ageing.

## Children and learning

Children are particularly vulnerable to the consequences of uncorrected refractive error. Children who do not receive adequate correction for their refractive error are at risk of lifelong visual impairment due to amblyopia. This not only affects the individual, but also stunts the development of entire communities and nations. In addition, uncorrected myopia and hyperopia can hinder academic progress, leading to lower educational attainment and future career opportunities. A myopic child who can't see the chalkboard may be misdiagnosed with learning disabilities.

## Productivity and economic impact

Whereas cataract, the other leading cause of avoidable visual impairment, affects mostly older, non-working people, the impact of refractive error extends throughout the working-age population. Correcting refractive error increases productivity (by up to 32%^5^) and reduces absenteeism and job losses. This results in substantial economic gains for individuals and nations. In low- and middle-income countries, where access to vision care is limited, the economic consequences of not treating individuals is especially severe. Uncorrected myopia leads to an estimated global productivity loss of US $244 billion^6^ while presbyopia may be responsible for a loss of between US $25 billion^7^ and US $54 billion.^8^

## Healthy ageing

Vision impairment has been associated with worse outcomes among older adults, including cognitive decline and dementia,^9^ depression,^10^ and increased risk of falls^11^ and fracture,^12^ all of which increase morbidity and mortality. Refractive services therefore have the potential to not only improve vision and quality of life, but also to save lives.

## Eye health equity

Access to eye care is often inequitable and vision impairment due to refractive error can make this worse, with rural and marginalised communities suffering the most. This is true both on a global scale and within communities. Globally, South Asia, South East Asia, and sub-Saharan Africa have the highest prevalence of uncorrected refractive error (standardised for age).^2^ Within communities, prevalence is higher, and willingness-to-pay (a measurement of how much a person can afford to spend) for spectacles is lower, among those with lower incomes.^13^,^14^ Addressing refractive error is not just a matter of vision; it is a matter of social justice. It is about ensuring that everyone has the same opportunities for education, employment, and a high quality of life. Neglecting refractive error can worsen inequalities and social exclusion.

## The role of refractive error care providers

Every eye care provider plays a crucial role in addressing this issue, whether on a local, regional, or global scale. Refractive service providers, usually led by optometrists, are the bridge between policy and practice: global health goals can only have a tangible impact if eye care workers are there to implement them.

Including eye care in general health care, and making spectacles for presbyopia available in the community (e.g., at pharmacies) is crucial given the scale of the problem; this is in line with WHO recommendations for the provision of presbyopia correction at the community level.^15^ Technology provides an opportunity to ease this transition by making it possible to train community and primary health care workers from a distance, provide decision support algorithms, and the potential for remote supervision or telemedicine input when needed. Tele-refraction is a growing field, although there is not yet enough evidence supporting its use.^16^

## Advocating for resources

Policy makers must prioritise uncorrected refractive error as a part of their broader health and development agenda. Investing in refractive error and optical services not only improves the lives of individuals, but also promotes economic development. Access to affordable spectacles and regular eye examinations should be integrated into national health systems.

**Figure:**
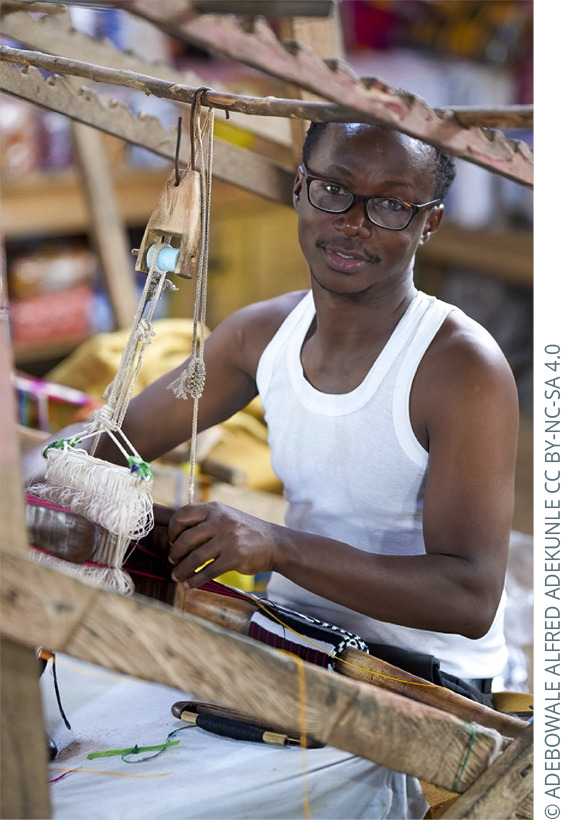
Spectacles for near vision improve productivity. ghana

## Promoting education and awareness

Refractive error care providers can help raise awareness about the importance of regular eye examinations and the availability of affordable corrective measures. They can also advocate for comprehensive school vision screening programmes to identify and address refractive error in children early, and they can offer workplace assessments for employees as a way to increase productivity and safety. Refractive error correction is also key to road safety: visual impairment has been found to be associated with a 46% greater risk of road traffic collision.^17^

## WHO SPECS 2030

In 2021, WHO member states endorsed a global target to increase effective refractive error coverage (eREC) by 40 percentage points. The World Health Organization has recently launched a new SPECS 2030 initiative, aiming to provide quality, affordable and people-centred refractive error services to everyone who needs them.

## Conclusion

Near and distance vision impairment, due to uncorrected refractive error and presbyopia, is a global health issue with profound implications for individuals, communities, and nations. Stakeholders and health workers at all levels have a role to play in tackling this large, but solvable, problem. As policy makers and health service managers, the responsibility lies with you to allocate resources and prioritise refractive services in national health agendas. As refractive error care providers, the work you do restores not only your patients’ eyesight, but also their education, income, and safety.

**Recent global estimates of refractive error and presbyopia**

These tables summarise some of the most widely accepted estimates of the magnitude of refractive error in recent years, and the studies on which these are based. Note that the Eliminating Poor Vision in a Generation Report uses a different threshold (visual acuity < 6/9), which has contributed to the large difference in reported magnitude. However, there remains a shortage of primary data on which to base estimates. Ongoing data collection via eye care programmes and surveys, such as Rapid Assessment of Avoidable Blindness (RAAB) surveys, should improve the accuracy of future estimates.

**Table 1:** Global estimates of the number of people with distance vision impairment or blindness due to refractive error (uncorrected, corrected, and total).

	Holden et al (2016)^18^	Eliminating Poor Vision in a Generation Report^19^	World Report on Vision^3^	Vision Loss Expert Group^2^ and Bourne et al (2020), Lancet Commission on Global Eye Health^1^
*Definitions*	*≤ -0.5 diopter*	*Visual acuity (VA) < 6/9*	*Moderate to severe visual impairment (MSVI) 3/60 < VA < 6/18*	*MSVI or blindness (VA < 6/18)*
Uncorrected refractive error	-	2.7 billion^16^^20^	123.7 million	161 million
Corrected refractive error	-	2.0 billion^16^^20^	-	-
Total refractive error	2.6 billion (myopia only, for 2020)	4.7 billion^16^^20^	-	-

**Table 2:** Global estimates of near vision impairment due to presbyopia.

	Fricke et al (2018)^4^	Eliminating Poor Vision in a Generation Report^19^	World Report on Vision^3^	Vision Loss Expert Group and Bourne et al (2020),^2^ Lancet Commission on Global Eye Health^1^
Uncorrected presbyopia	826 million (for 2015)	-	826 million^4^	510 million (for 2020) 866 million (predicted for 2050)
Corrected presbyopia	1 billion	-	1 billion^4^	-
Total presbyopia	1.8 billion (2015)	1.4 billion^19^ (with no other refractive error)	1.8 billion^4^	-
